# Anticipatory control of human gait following simulated slip exposure

**DOI:** 10.1038/s41598-020-66305-1

**Published:** 2020-06-15

**Authors:** Sander B. Swart, Rob den Otter, Claudine J. C. Lamoth

**Affiliations:** University of Groningen, University Medical Centre Groningen, Department of Human Movement Sciences, Groningen, The Netherlands

**Keywords:** Motor control, Biomechanics

## Abstract

A cautious gait (CG), marked by wider and shorter steps, is typically employed to mitigate expected perturbations proactively. However, it is not well understood *if* and *how* CG is informed by the task requirements. Therefore, we assessed *how* CG is adjusted to these requirements. Three groups of ten healthy young adults were exposed to a single uninterrupted protocol of treadmill walking that consisted of three distinct phases. Spatiotemporal step characteristics and margins of stability of the unperturbed strides were compared when participants were (i) only warned of a perturbation, (ii) exposed to fifty unilateral (right) slip-like perturbations and (iii) kept unaware of perturbation removal. Only the perturbation intensity predictability differed between groups. This was either kept consistent or pseudo-randomly or randomly varied. Participants walked with wider and shorter steps following the perturbation warning. However, this extinguished in continuing perturbation absence. Next, during perturbation exposure, participants shortened the step of the perturbed but increased the step of the unperturbed leg. This did not differ between groups. Finally, participants persisted in displaying CG on perturbation removal, but this extinguished over time. Collectively, we show that CG is functionally adjusted to the task requirements. These findings may have practical implications for fall-prevention training.

## Introduction

Bipedal gait is a challenging activity, considering that the vertical projection of the centre of mass (CoM) is outside the base of support (BoS) for approximately 80% of the gait cycle^1^. In fact, walking involves continuous falling motions, which are caught by a relocation of the BoS during every step. Nevertheless, walking is generally stable and adaptive, as healthy adults are able to adjust their gait continuously to accommodate environmental and task demands^2^. However, walking in an environment encountering unpredictable or changing events becomes particularly challenging when the walking performance declines due to natural ageing or pathology^3^. As a result, older adults often employ a cautious gait (CG) strategy to safeguard against potential balance loss^4,5^. Although this form of anticipatory control has been described as a generic strategy that is characterized by wider and shorter steps^6^, it is not well understood *if* and *how* CG is informed by the requirements of the task. The present study addressed *how* anticipatory control of gait is adjusted to accommodate these requirements.

Anticipatory control relies on sensory input and provides the means to identify and proactively accommodate environmental challenges instantaneously^2^. Walking on ice is a simple illustration of this, which is typically characterized by wider and shorter steps. These cautious adjustments safeguard dynamic balance and can reduce the need for time-critical, reactive control^7^. Putatively, people employ a CG by the necessity of uncertainty (i.e. where, when, and if a perturbation will occur). This is substantiated by empirical studies, which show that young adults spontaneously employ a CG pattern when they are being blindfolded^8,9^. In addition, experience with the task and its requirements may result in a reduction of uncertainty. This may alter how anticipatory control is implemented. For instance, the first exposure to an unexpected balance perturbation is known to generate exaggerated postural reactions^10^. However, when the same perturbation is presented repeatedly, this first trial reaction attenuates swiftly (i.e. the first trial effect). Although many task features may affect the degree to which expectation influences the response, anticipatory control generally becomes more prominent through experience with perturbing stimuli^7^. Arguably, a reduction of uncertainty through repeated exposure to perturbing stimuli promotes anticipatory control.

Besides blindfolding people, a CG can be induced in young adults by merely warning them of a potential slip hazard. Even without prior simulated slip experience young adults proactively reduce their stride length (~11%)^11^ and flatten their foot at touch down (~20%)^12^ when they approach a pseudo-slippery floor. Similar results, such as a step length reduction of ~2% following a slip warning have been reported within time-confined trials (i.e. 30 sec.) of treadmill walking at a fixed gait speed of 1.2 m/s^13^. Despite an actual absence of a perturbation task and a marginally informed condition (i.e. warning of a perturbation), these studies show that the expectation of an upcoming perturbation induces anticipatory adjustments, which are considered features of a CG.

However, behavioural experience with the perturbation task might be needed to adjust anticipatory control with task requirements^12,14^. After exposure to a consistent slip perturbation, more pronounced anticipatory adjustments have been reported, e.g. ~2% smaller peak vertical forces, ~20% lower peak braking shear forces^12^ and a larger inclined trunk angle^13^. Presenting the perturbation consistently in terms of its nature (slip or trip), side (left or right) or intensity (duration in sec.) may thus increase the predictability of the task requirements and promote anticipatory control. Yet, features of perturbation events in daily life are rarely consistent or predictable. Therefore, in addition to the putative role of anticipatory control for consistent gait perturbations, it is important to understand *if* and *how* inconsistency in perturbations can be utilized to inform anticipatory control. Since such inconsistency may elevate the perceived risk of balance loss, it is conceivable that unpredictable perturbations elicit more pronounced anticipatory adjustments.

The extent to which simulated perturbations affect the dynamic balance of CG can be quantified in terms of feasibility regions^15^ or margins of stability (MoS)^16^. These methods are based on the inverted pendulum model of walking. In this model, bipedal gait is conceptualized as an inverted pendulum, in which the pendulum and a single point mass on top, represent the stance leg and human body, respectively^1^. To describe its motion state, both the position and the velocity of the CoM must be accounted for^16^. This concept, known as the extrapolated CoM (XCoM), can be used to operationalize a condition for dynamic balance by assessing its minimal distance to the centre of pressure (CoP). While a positive MoS suggests a stable configuration, a negative MoS corresponds to an unstable configuration. Empirical findings have shown that this margin can be increased in the frontal plane by taking wider steps^17^. Similarly, in the sagittal plane, a shorter step decreases the MoS^17^, as the leading foot (i.e. BoS) is placed less anterior. This spatial adjustment has been shown to enhance stability against backward loss of balance^18,19^. Hence, by taking wider and shorter steps, people intend to prepare for – and minimize the consequences of – mediolateral (ML) and backward loss of balance, respectively.

Since the properties of upcoming perturbations can be inconsistent, a better understanding of anticipatory control requires a detailed articulation of *if* and *how* features of CG, such as spatiotemporal step characteristics and dynamic balance, are adjusted to accommodate these task requirements. Therefore, the primary aim of the current study was to assess *if* and *how* people adjust spatiotemporal step characteristics and MoS to accommodate the requirements of a gait perturbation task. To this end, participants were exposed to a single uninterrupted protocol consisting of 3 distinct phases. Spatiotemporal step characteristics and the MoS of unperturbed strides were compared when participants were (i) only warned of a perturbation, (ii) exposed to 50 unilateral (right) slip-like perturbations and (iii) kept unaware of perturbation removal. Three groups were tested. Only the predictability of the perturbation intensity differed between groups, which was either varied pseudo-randomly or randomly or kept consistent. A motorized treadmill was utilized as it enables to control for potentially confounding effects of gait speed on spatial features of CG^13,20^ and to assess gait parameters over a longer period. We hypothesized that young adults adjust their gait purposefully to accommodate the consistent side and nature of the perturbation. This will result in increased stability against backward loss of balance and smaller step lengths and single support times compared to the baseline and warning phases. In addition, we hypothesize that anticipatory adjustments are more pronounced when the predictability of the perturbation intensity is random or pseudo-random compared to consistent.

## Materials and Methods

### Participants

A convenience sample of 30 healthy young adults was recruited in this study (17 males, 13 females, 21.6 ± 2.2 years old, body mass: 70.1 ± 9.8 kg, body height: 1.78 ± 0.085 m, leg length: 0.92 ± 0.039 m), based on similar studies in the field of balance control during perturbed gait^21,22^. Participants were excluded if they had any prior experience with slip-like perturbation experiments or if they had any neurological, cognitive, or orthopaedic impairments that could affect the outcome of the experiment. All procedures were approved by the Local Ethics Committee of the Department of Human Movement Sciences (LTc201800978) University Medical Centre Groningen (UMCG), the Netherlands (NL) and were in accordance with the principles outlined in the Declaration of Helsinki^23^. All participants gave their written informed consent prior to the experiment.

### Experimental procedures

**Figure 1:**
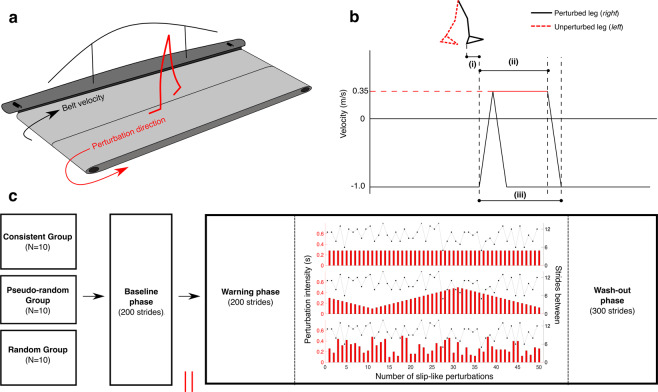
Overview of the experimental paradigm. (**a**) Perturbation method, participants walked on a split-belt treadmill at a velocity of -1 m/s (black arrow), at perturbation onset both belts accelerated to a velocity of 0.35 m/s (red arrow). (**b**) Perturbations were (i) induced shortly after the right foot heel strike (Supplementary Table S1). Perturbation profile, the intensity of the perturbation (ii) was either randomly or systematically varied between 0.1 and 0.5 seconds (red solid line), (iii) represents the total perturbation intensity (Supplementary Table S1). (**c**) Experimental paradigm, the experiment consisted of four phases: the baseline, perturbation warning, perturbation, and wash-out phase. Only the perturbation phase differed between experimental groups. The treadmill was only stopped after the baseline (red vertical lines), subsequently a predefined protocol commenced, without manual interference.

A split-belt treadmill (M-gait, Motekforce Link, Amsterdam, NL) was used in this study to induce slip-like perturbations during gait (Fig. 1a). At perturbation onset (Fig. 1b), both belts accelerated simultaneously in the forward direction shortly after the instance of right foot strike (Supplementary Table S1), resulting in a forward displacement of the BoS relative to the CoM. The belts accelerated to a velocity of 0.35 m/s (Fig. 1b) and returned to normal walking speed (−1.0 m/s) when the perturbation intensity matched the imposed intensity of the particular experimental group (Fig. 1c) (i.e. consistent, pseudo-random or random).

The intensity of the slip perturbations was varied in this study. Larger slip distances have been shown to elicit larger postural disturbances during movable platforms slips compared to short distances^24^. Therefore, the perturbation intensity was varied in this study by altering the duration of the slip perturbation. The maximal intensity was set at 0.5 sec., which was found to be sufficiently challenging without causing real falls.

To examine the effect of the perturbation consistency, the intensity of subsequent perturbations was varied in a consistent, pseudo-random or random fashion (Fig. 1c). The consistent group (N = 10) received perturbations of identical intensity (0.28 sec.). Secondly, the pseudo-random group (N = 10) received perturbations in which the intensity either increased or decreased with 0.02 sec. compared to its antecedent. Lastly, the random group (N = 10) received the same perturbations as the pseudo-random group but presented randomly. This implies that the intensities of subsequent perturbations were not correlated (Supplementary Table S1). The intensity of the first perturbation was approximately (due to randomization procedures) similar for the consistent group (i.e. 0.28 sec.), pseudo-random group (i.e. 0.30 sec.) and random group (i.e. 0.26 sec.). Participants were exposed to a total of 50 perturbations. The average intensity of these 50 perturbations was identical between experimental groups (i.e. 0.28 sec.). The number of strides between perturbations was randomly varied between 5–14 strides (Fig. 1c) and was kept the same between groups.

### Protocol

Participants were assigned to one of the experimental groups in an unstructured fashion. Safety instructions were given before experimentation. Subsequently, participants were fitted in a safety harness that did not support their body weight during the experiment and they were instructed to walk with one foot on each belt. The protocol consisted of four phases: (i) the baseline phase, (ii) the warning phase, (iii) the perturbation phase and (iv) the wash-out phase (Fig. 1c). During the baseline phase, participants walked on the treadmill (v = −1 m/s) for 210 strides without getting perturbed. This phase was included to make sure that the participants got acquainted with walking on the split-belt treadmill and served as a control phase to which other experimental phases were compared. After the baseline phase, the treadmill was stopped. Participants were told that from this point on they could experience a slip perturbation. The instructions were delivered identically for all participants. Participants were informed that if they experience a slip, they should try to re-establish their balance and keep on walking and only use the handrail when they really thought they would fall. Next, after 205 strides, the perturbation phase commenced without the participants’ knowledge. In total, 50 slip-like perturbations were presented unilaterally to the right leg in the perturbation phase. After this phase, participants walked for an additional 310 strides during the wash-out phase without being informed that the perturbations exposure had ended.

### Data collection

Anthropometric characteristics, including leg length, body height, and body weight were measured before conducting the experiment. During the experiment, 3D ground reaction forces (GRF) (N) and moments (of force) (Nm) were measured using two separate force plates embedded underneath each belt and simultaneously recorded with D-flow software (Motekforce Link, Amsterdam, NL, high-performance mode) at a sampling frequency of 300 Hz.

### Data analysis

The data was analysed using custom-made Matlab (version r2016b; The Mathworks Inc., Natick, MA) routines. An XYZ coordinate system was used, with the x-axis in the anteroposterior (AP) direction, y-axis in the vertical direction and z-axis in the ML direction^25^. Gait events were defined as the moments where the vertical GRF (GRF_y_) on the individual force plates crossed the threshold of 50 N. This process was checked visually to identify missed or double gait events. GRFs and moments were filtered using a 2nd order low-pass Butterworth filter (cf = 15 Hz). CoP position was calculated for each force plate in the x and z direction using Eqs. 1 and 2.

1 $${{\rm{CoP}}}_{{\rm{x}}}=-\frac{{{\rm{M}}}_{{\rm{z}}}}{{{\rm{F}}}_{{\rm{y}}}}$$

2 $${{\rm{CoP}}}_{{\rm{z}}}=\frac{{{\rm{M}}}_{{\rm{x}}}}{{{\rm{F}}}_{{\rm{y}}}}$$

in which M represents the moment of force (Nm) and F the force (N). CoP data of the individual belts were combined to ensure continuous monitoring of both CoP and CoM position. This combined simulated CoP position was calculated in both the x and z direction by scaling the CoP position of each force plate to the magnitude of its GRF_y_ using Eq. 3.

3 $${\rm{CoP}}=\frac{{{\rm{GRF}}}_{{\rm{y}}({\rm{L}})}\times {{\rm{CoP}}}_{({\rm{L}})}+{{\rm{GRF}}}_{{\rm{y}}({\rm{R}})}\times {{\rm{CoP}}}_{({\rm{R}})}}{{{\rm{GRF}}}_{{\rm{y}}({\rm{L}})}+{{\rm{GRF}}}_{{\rm{y}}({\rm{R}})}}$$

The combined CoP position was used for all further analyses. Step lengths (m) were calculated using Eq. 4 and defined as the difference in CoP_x_ position (m) between left and right foot at heel strike, normalized for leg length (l/l_0_)^26^.

4 $${\rm{Step}}\,{{\rm{length}}}_{{\rm{L}},{\rm{R}}}=|{{\rm{CoP}}}_{{\rm{x}}}({{\rm{HS}}}_{{\rm{L}},{\rm{R}}})-({{\rm{TfD}}}_{{\rm{R}},{\rm{L}}}+{{\rm{CoP}}}_{{\rm{x}}}{({\rm{TO}}}_{{\rm{R}},{\rm{L}}}))|$$

in which the travelled foot distance (TfD) (m) is the time between contralateral toe-off and heel strike, multiplied by the belt speed. The virtual foot position was calculated by adding the travelled foot distance (TfD) and CoP_x_ position of the contralateral leg. Spatiotemporal symmetry indexes were calculated to assess whether anticipatory control differed between perturbed and unperturbed leg using Eqs. 5 and 6 ^27^.

5 $${\rm{S}}{\rm{t}}{\rm{e}}{\rm{p}}\,{\rm{l}}{\rm{e}}{\rm{n}}{\rm{g}}{\rm{t}}{\rm{h}}\,{\rm{s}}{\rm{y}}{\rm{m}}{\rm{m}}{\rm{e}}{\rm{t}}{\rm{r}}{\rm{y}}({i})=\frac{{\rm{s}}{\rm{t}}{\rm{e}}{\rm{p}}\,{{\rm{l}}{\rm{e}}{\rm{n}}{\rm{g}}{\rm{t}}{\rm{h}}}_{{\rm{L}}}({i})-{\rm{s}}{\rm{t}}{\rm{e}}{\rm{p}}\,{{\rm{l}}{\rm{e}}{\rm{n}}{\rm{g}}{\rm{t}}{\rm{h}}}_{{\rm{R}}}({i})}{{\rm{s}}{\rm{t}}{\rm{e}}{\rm{p}}\,{{\rm{l}}{\rm{e}}{\rm{n}}{\rm{g}}{\rm{t}}{\rm{h}}}_{{\rm{L}}}(i)+{\rm{s}}{\rm{t}}{\rm{e}}{\rm{p}}\,{{\rm{l}}{\rm{e}}{\rm{n}}{\rm{g}}{\rm{t}}{\rm{h}}}_{{\rm{R}}}({i})}$$

Single support symmetry for every step *i* was calculated to reflect on temporal aspects of gait throughout the experiment, using Eq. 6 [adapted from^27^].

6 $${\rm{S}}{\rm{i}}{\rm{n}}{\rm{g}}{\rm{l}}{\rm{e}}\,{\rm{s}}{\rm{u}}{\rm{p}}{\rm{p}}{\rm{o}}{\rm{r}}{\rm{t}}\,{\rm{s}}{\rm{y}}{\rm{m}}{\rm{m}}{\rm{e}}{\rm{t}}{\rm{r}}{\rm{y}}(i)=\frac{{\rm{s}}{\rm{i}}{\rm{n}}{\rm{g}}{\rm{l}}{\rm{e}}\,{\rm{s}}{\rm{u}}{\rm{p}}{\rm{p}}{\rm{o}}{\rm{r}}{\rm{t}}\,{{\rm{t}}{\rm{i}}{\rm{m}}{\rm{e}}}_{{\rm{L}}}(i)\,-{\rm{s}}{\rm{i}}{\rm{n}}{\rm{g}}{\rm{l}}{\rm{e}}\,{\rm{s}}{\rm{u}}{\rm{p}}{\rm{p}}{\rm{o}}{\rm{r}}{\rm{t}}\,{{\rm{t}}{\rm{i}}{\rm{m}}{\rm{e}}}_{{\rm{R}}}({i})}{{\rm{s}}{\rm{i}}{\rm{n}}{\rm{g}}{\rm{l}}{\rm{e}}\,{\rm{s}}{\rm{u}}{\rm{p}}{\rm{p}}{\rm{o}}{\rm{r}}{\rm{t}}\,{{\rm{t}}{\rm{i}}{\rm{m}}{\rm{e}}}_{{\rm{L}}}(i)+{\rm{s}}{\rm{i}}{\rm{n}}{\rm{g}}{\rm{l}}{\rm{e}}\,{\rm{s}}{\rm{u}}{\rm{p}}{\rm{p}}{\rm{o}}{\rm{r}}{\rm{t}}\,{{\rm{t}}{\rm{i}}{\rm{m}}{\rm{e}}}_{{\rm{R}}}({i})}$$

in which the single support time (s) is defined as the time between contralateral toe-off and contralateral heel strike. Step width (m) was calculated for every step *i* throughout the experiment using Eq. 7.

7 $${\rm{S}}{\rm{t}}{\rm{e}}{\rm{p}}\,{\rm{w}}{\rm{i}}{\rm{d}}{\rm{t}}{\rm{h}}({i})=|{\rm{C}}{\rm{o}}{\rm{P}}{(j)}_{{\rm{z}}}^{min\,,\,max\,}-{\rm{C}}{\rm{o}}{\rm{P}}{(k)}_{{\rm{z}}}^{max\,,\,min}|$$

in which the absolute difference between local minimum/maximum CoP_z_ position during ipsilateral single support phase *j* and the local maximum/minimum CoP_z_ position during the consecutive contralateral single support phase *k* was determined^28^.

Dynamic balance was quantified using the MoS and was calculated for both legs in AP and ML direction at contralateral toe-off^16^. The fusion integration method from Schepers *et al*.^29^ was used to calculate the XCoM, several steps were taken to achieve this. Firstly, the CoM position was calculated by integrating the CoM acceleration (GRF_x,z_ divided by body weight) twice. Subsequently, the CoM_x,z_ position was high-pass filtered using a 2nd order Butterworth (cf = 0.2 Hz) to prevent integration induced drift^29^. The absolute CoM_x,z_ position was acquired by adding the low-pass filtered (2nd order Butterworth, cf = 0.2 Hz) CoP_x,z_ signal. Finally, the XCoM position (m) was calculated using Eq. 8.

8 $${\rm{XCoM}}({\rm{x}},\,{\rm{z}})={\rm{CoM}}({\rm{x}},\,{\rm{z}})+\frac{{\rm{Co}}{\rm{M}}{\prime} ({\rm{x}},\,{\rm{z}})}{\surd \frac{{\rm{g}}}{{\rm{l}}}}$$

in which CoM^′^ is the velocity of the CoM (m/s) in the x or z direction, *g* (9,81 m/s^2^) is the gravitational constant and *l* is the leg length (m), defined as greater trochanter height multiplied by 1.2 in the frontal and 1.34 in the sagittal plane^16^. Lastly, the MoS (m) was defined as the distance between CoP_x,z_ position and XCoM_x,z_ position at contralateral toe-off *j* for every step *i* using Eq. 9 ^16^.

9 $${{\rm{M}}{\rm{o}}{\rm{S}}}_{{\rm{x}},{\rm{z}}}(i)={{\rm{C}}{\rm{o}}{\rm{P}}}_{{\rm{x}},{\rm{z}}}(j)-{{\rm{X}}{\rm{C}}{\rm{o}}{\rm{M}}}_{{\rm{x}},{\rm{z}}}({j})$$

Since the acceleration and deceleration of the treadmill at the experiment start might impose confounding effects^13^, the first 5 strides of the baseline and warning phase and the last 5 strides of baseline and wash-out phase were excluded from the analysis. Thus 200 strides from the baseline and warning phase and 300 strides from the wash-out phase were analysed.

Next, all dependent variables were analysed and averaged over the first 20 strides of the warning and wash-out phase and the last 20 strides of the baseline, warning, and wash-out phase. This resulted in the following phases: late baseline (LB), early anticipation (EA), late anticipation (LA), early wash-out (EW), and late wash-out (LW).

Analysis of the perturbation phase was restricted to the unperturbed strides in-between perturbations. Since recovery from moderate slip perturbation takes 2 to 3 steps^30^, perturbed steps were defined as perturbation stride and subsequent two recovery strides. Next, all dependent variables were averaged over the unperturbed strides between perturbation 1 to 5 (early perturbation; EP) and 46 to 50 (late perturbation; LP). Finally, the beginning of the wash-out phase was defined as the moment when 5 recovery strides were taken after the last perturbation.

### Statistical analysis

The dependent variables, step length symmetry, single support symmetry, normalized step length_R,L_, normalized single support time_R,L_, AP MoS_R,L_ and ML MoS_R,L_ were analysed during LB, EA, LA, EP, LP, EW and LW. To test for differences between the experimental phases, repeated measures (RM) ANOVA’s were performed for each dependent variable. The within factor PHASE had 7 levels (LB, EA, LA, EP, LP, EW, LW) and the between factor GROUP had 3 levels (consistent, pseudo-random, random) in the 11 respective RM ANOVA’s. Main effects of the factor PHASE were further analysed with simple contrasts, which compared each experimental phase with LB. This was done to determine whether the dependent variables during the experimental phases differed from normal walking. Additionally, these contrast comparisons were compared between experimental groups, to check whether the phase interactions differed between experimental groups.

Statistical significance was set at an alpha of 5% for all analyses. Bonferroni corrections for multiple comparisons were applied to the p-values of the main and post-hoc analysis. Sphericity violations were corrected using a Greenhouse-Geisser correction. Statistical analysis was performed using SPSS (IBM Corp. Released 2015. IBM SPSS Statistics for Windows, Version 23.0. Armonk, NY: IBM Corp.).

## Results

No significant main effects of the between factor GROUP were found (Table 1). Significant main effects of the within factor PHASE were found for all dependent variables (Table 1).

**Table 1:** Main and interaction effects.

Variable	Factor	ε	F (*d.f.)*	P	ηp^2^
Step Length Right	PHASE	0.54	23.08 (*3.21,86.68*)	<0.001^*^	0.46
	GROUP * PHASE	0.54	1.00 (*6.42,86.68*)	0.99	0.07
Step Length Left	PHASE	0.51	23.40 (*3.08,83.02*)	<0.001^*^	0.46
	GROUP * PHASE	0.51	1.05 (*6.15,83.02*)	0.99	0.07
Single Support Right	PHASE	0.65	9.16 (*3.89,104.95*)	<0.001^*^	0.25
	GROUP * PHASE	0.65	1.72 (*7.77,104.95*)	0.99	0.11
Single Support Left	PHASE	0.46	18.41 (*2.77,74.86*)	<0.001^*^	0.41
	GROUP * PHASE	0.46	0.69 (*5.55,74.86*)	0.99	0.05
Step Length Symmetry	PHASE	0.54	18.63 (*3.25,87.68*)	<0.001^*^	0.41
	GROUP * PHASE	0.54	1.13 (*6.50,87.68*)	0.99	0.08
Single Support Symmetry	PHASE	0.58	27.17 (*3.46,93.38*)	<0.001^*^	0.50
	GROUP * PHASE	0.58	1.47 (*6.92,93.38*)	0.99	0.10
Step Width	PHASE	0.65	40.020 (*3.90,105.38*)	<0.001^*^	0.60
	GROUP * PHASE	0.65	0.93 (*7.81,105.38*)	0.99	0.064
Margin of Stability Right (AP)	PHASE	0.57	38.57 (*3.44,92.95*)	<0.001^*^	0.59
	GROUP * PHASE	0.57	1.52 (*6.89,92.95*)	0.99	0.10
Margin of Stability Left (AP)	PHASE	0.54	19.91 (*3.24,87.53*)	<0.001^*^	0.42
	GROUP * PHASE	0.54	1.06 (*6.48,87.53*)	0.99	0.07
Margin of Stability Right (ML)	PHASE	0.70	26.29 (*4.18,112.84*)	<0.001^*^	0.49
	GROUP * PHASE	0.70	0.58 (*8.36,112.84*)	0.99	0.04
Margin of Stability Left (ML)	PHASE	0.53	41.76 (*3.18,85.87*)	<0.001^*^	0.61
	GROUP * PHASE	0.53	0.67 (*6.36,85.87*)	0.99	0.047

^*^Indicates significance at an alpha of 5% for Bonferroni corrected p-value.

Violations of sphericity were corrected using Greenhous-Geisser estimate (ε).

ML – mediolateral, AP – anteroposterior.

Significant main effects of the within factor PHASE were further analysed with simple contrasts (Table 2).

**Table 2:** Post hoc analysis of the PHASE comparison.

Variable	Post hoc comparison											
	LB vs EA		LB vs LA		LB vs EP		LB vs LP		LB vs EW		LB vs LW	
	F_(1,27)_; p-value	ES	F_(1,27)_; p-value	ES	F_(1,27)_; p-value	ES	F_(1,27)_; p-value	ES	F_(1,27)_; p-value	ES	F_(1,27)_; p-value	ES
SL_R_	48.40; <0.001^*^	0.74	0.045; 0.99	0.02	76.42; <0.001^*^	0.97	11.00; 0.018^*^	0.44	7.73; 0.060	0.43	7.66; 0.060	0.32
SL_L_	13.21; 0.006^*^	0.50	0.98; 0.99	0.11	8.46; 0.042^*^	0.50	24.11; <0.001^*^	0.66	18.41; <0.001^*^	0.57	26.21; <0.001^*^	0.53
SST_R_	11.84; 0.012^*^	0.32	0.24; 0.99	0.05	12.71; 0.006^*^	0.45	5.86; 0.14	0.31	2.096; 0.96	0.21	3.40; 0.46	0.23
SST_L_	23.89; <0.001^*^	0.49	0.31; 0.99	0.06	12.52; 0.006^*^	0.36	10.29; 0.018^*^	0.38	5.78; 0.14	0.36	8.96; 0.036^*^	0.40
SLS	3.81; 0.37	0.32	2.09; 0.96	0.24	8.04; 0.054	0.70	42.59; <0.001^*^	1.62	38.34; <0.001^*^	1.60	5.28; 0.18	0.44
SSS	4.54; 0.25	0.44	0.003; 0.99	0.01	4.051; 0.32	0.50	36.94; <0.001^*^	1.40	31.18; <0.001^*^	1.29	3.25; 0.50	0.43
SW	10.08; 0.024^*^	0.19	11.05; 0.018^*^	0.23	4.93; 0.21	0.18	24.66; <0.001^*^	0.38	27.20; <0.001^*^	0.41	80.96; <0.001^*^	0.59
AP MoS_R_	10.41; 0.018^*^	0.40	0.63; 0.99	0.09	68.07; <0.001^*^	1.23	39.92; <0.001^*^	1.13	28.08; <0.001^*^	1.01	5.73; 0.14	0.31
AP MoS_L_	9.69; 0.024^*^	0.48	5.44; 0.16	0.28	0.088; 0.99	0.06	48.42; <0.001^*^	0.80	23.29; <0.001^*^	0.68	30.15; <0.001^*^	0.61
ML MoS_R_	1.11; 0.99	0.08	14.33; 0.006^*^	0.24	16.32; <0.001^*^	0.41	6.14; 0.12	0.21	5.42 0.17	0.21	65.96; <0.001^*^	0.57
ML MoS_L_	13.42; 0.006^*^	0.25	6.32; 0.11	0.17	17.29; <0.001^*^	0.44	35.73; <0.001^*^	0.44	23.85; <0.001^*^	0.39	53.45; <0.001^*^	0.53

Simple contrasts between late baseline (LB) vs early anticipation (EA), LB vs late anticipation (LA), LB vs early perturbation (EP), LB vs late perturbation (LP), LB vs early wash-out (EW) and LB vs late wash-out (LW) were performed for all dependent variables.

^*^Indicates significance at an alpha of 5% for Bonferroni corrected p-value.

ES – effect size (Cohen’s d).

SL_R,L_ – step length, SST_R,L_ – single support time, SLS – step length symmetry, SSS – single support symmetry, SW – step width, AP MoS_R,L_ – anteroposterior margin of stability, ML MoS_R,L_ – mediolateral margin of stability.

### *Baseline phase vs. Warning phase* – The effect of a perturbation warning on anticipatory locomotor control

**Figure 2:**
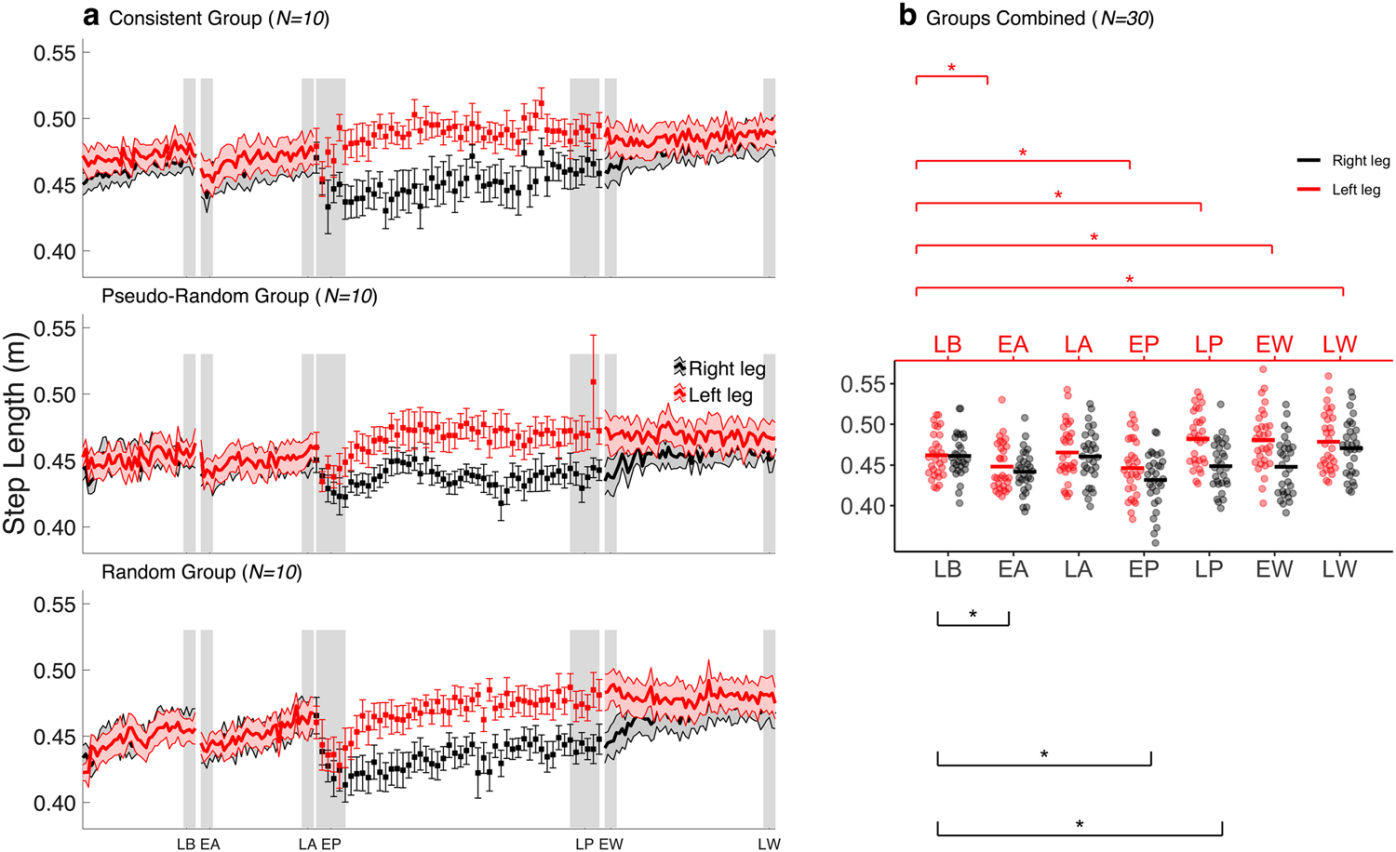
Step length during the experiment. (**a**) Normalized step length^26^ during the experiment is shown for the consistent (top) (N = 10), the pseudo-random (middle) (N = 10) and the random group (bottom) (N = 10). Left and right step lengths were averaged in bins of 5 strides during the baseline, warning, and wash-out phase. During the perturbation phase, the left and right step lengths of the unperturbed strides between two consecutive perturbations were averaged. Shaded areas and whiskers around the mean represent the standard error. Grey shaded blocks represent the different phases throughout the experiment: late baseline (LB), early anticipation (EA), late anticipation (LA), early perturbation (EP), late perturbation (LP), early wash-out (EW) and late wash-out (LW). (**b**) Individual data points of all participants (N = 30) during the experimental phases. Black and red horizontal lines, respectively, represent the group mean. Significant post hoc comparisons for the within factor PHASE are shown with the asterisk.

Respective EA and LA during the warning phase were compared with LB, to assess CG without prior slip experience. The averaged results of the consistent group, pseudo-random group, random group, and the combined sample for step length are shown in Fig. 2. Detailed statistics are presented in Table 2. Participants significantly decreased their step length_R,L_ during EA compared to LB (Fig. 2b). Similarly, participants significantly reduced their left and right single support times during EA compared to LB. Accordingly, step length and single support symmetry were not significantly different from LB during EA. Gait during EA was further characterized by significantly increased step width compared to LB. In the continuing absence of a perturbation, participants increased their step length (Fig. 2a) and single support time. As such, bilateral step length, single support time, and its symmetry indices were not significantly different from LB during LA. During the warning phase, step width decreased and was significantly lower during LA compared to LB. These results indicate that participants temporarily adopt a CG following a warning.

**Figure 3:**
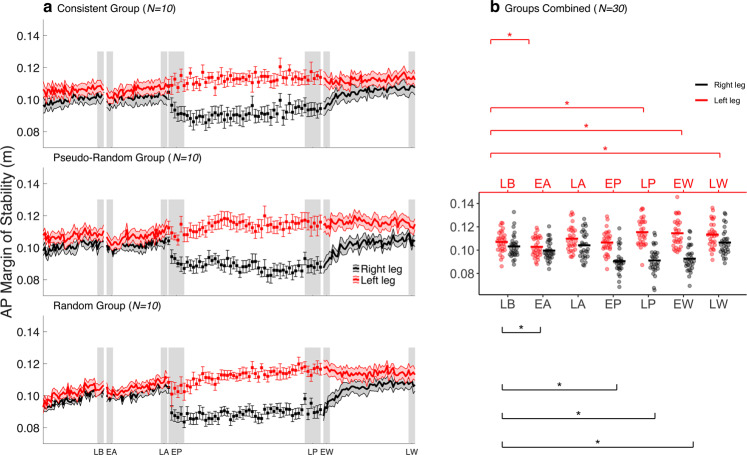
Anteroposterior (AP) margin of stability (MoS) at contralateral toe-off during the experiment. (**a**) AP MoS during the experiment is shown for the consistent (top) (N = 10), the pseudo-random (middle) (N = 10) and the random group (bottom) (N = 10). Left and right AP MoS were averaged within bins of 5 strides during the baseline, the warning, and the wash-out phase. During the perturbation phase, the left and right AP MoS of the unperturbed strides between two consecutive perturbations were averaged. Shaded areas and whiskers around the mean represent the standard error. Grey shaded blocks represent the different phases throughout the experiment: late baseline (LB), early anticipation (EA), late anticipation (LA), early perturbation (EP), late perturbation (LP), early wash-out (EW) and late wash-out (LW). (**b**) Individual data points of all participants (N = 30) during the experimental phases. Black and red horizontal lines, respectively, represent the group mean. Significant post hoc comparisons for the within factor PHASE are shown with the asterisk.

**Figure 4:**
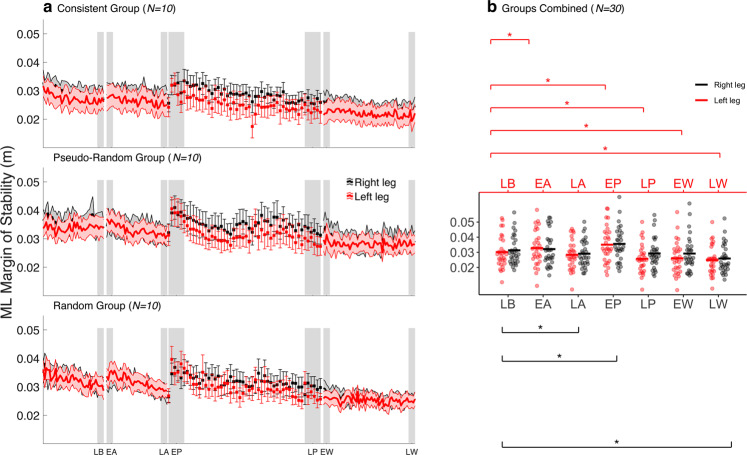
Mediolateral (ML) margin of stability (MoS) at contralateral toe-off during the experiment. (**a**) ML MoS during the experiment is shown for the consistent (top) (N = 10), the pseudo-random (middle) (N = 10) and the random group (bottom) (N = 10). Left and right ML MoS were averaged within bins of 5 strides during the baseline, the warning, and the wash-out phase. During the perturbation phase, the left and right ML MoS of the unperturbed strides between two consecutive perturbations were averaged. Shaded areas and whiskers around the mean represent the standard error. Grey shaded blocks represent the different phases throughout the experiment: late baseline (LB), early anticipation (EA), late anticipation (LA), early perturbation (EP), late perturbation (LP), early wash-out (EW) and late wash-out (LW). (**b**) Individual data points of all participants (N = 30) during the experimental phases. Black and red horizontal lines, respectively, represent the group mean. Significant post hoc comparisons for the within factor PHASE are shown with the asterisk.

The MoS was assessed in the frontal and sagittal planes, to quantify the dynamic balance of gait during the warning phase. The averaged results of the consistent group, pseudo-random group, random group, and the combined sample for AP MoS and ML MoS are shown in Figs. 3 and 4, respectively; statistics are presented in Table 2. The AP MoS_R,L_ was significantly decreased during EA compared to LB. In the frontal plane, the ML MoS_R,L_ increased during the EA compared to LB (Fig. 4b), but only significantly for ML MoS_L_. Over the course of the warning phase, AP MoS_R,L_ increased (Fig. 3a), as it was no longer significantly different from LB during LA (Fig. 3b). The ML MoS_R,L_ decreased throughout the warning phase (Fig. 4a). While ML MoS_R_ was significantly lower, ML MoS_L_ was not significantly different from LB during LA (Fig. 4b). Collectively, these findings show that participants temporarily increase their stability against backward loss of balance following a slip perturbation warning.

### *Baseline phase vs. Perturbation phase* – The effect of unilaterally presented slip-like perturbations on anticipatory control of gait

CG after perturbation exposure was assessed by comparing the unperturbed strides during EP and LP with LB. Table 2 presents detailed statistics. The participants significantly decreased their step length_R,L_ during EP compared to LB (Fig. 2b). Similarly, initial perturbation exposure resulted in reduced single support time_R,L_ during EP compared to LB. Furthermore, the step width was higher during EP compared to LB, but not significantly. Although the initial decrease in step length during EP gradually increased throughout the perturbation phase (Fig. 2a), this was evidently different between the left and the right step length. That is, step length_L_ was significantly higher, while step length_R_ was significantly lower during LP compared to LB (Fig. 2b). Similarly, single support time_L_ was significantly higher and single support time_R_ was considerably lower during LP compared to LB. Since the step length and single support time were different between perturbed and unperturbed leg during LP, spatiotemporal asymmetry occurred during LP compared to LB. Additionally, participants decreased their step width throughout the perturbation phase. As such, step width was significantly lower during LP compared to LB. Conjointly, the results show that participants adjust their gait to accommodate the perturbation requirements through reducing the step length and single support time of the perturbed leg and increasing the step length and single support time of the unperturbed leg.

During EP, the AP MoS_R_ was significantly decreased compared to LB, whereas AP MoS_L_ showed no significant difference with LB during EP (Fig. 3b). A significant increase for ML MoS_R,L_ was found during EP compared to LB (Fig. 4b). While the AP MoS_L_ increased, the AP MoS_R_ decreased throughout the perturbation phase (Fig. 3a). The AP MoS_L_ was significantly higher, and the AP MoS_R_ was significantly lower during LP than LB (Fig. 3b). The ML MoS decreased throughout the perturbation phase, with the ML MoS_R_ remaining slightly, but consistently, higher than the ML MoS_L_ (Fig. 4a). The ML MoS_L_ was significantly lower during LP than LB, whereas ML MoS_R_ was not significantly different compared to LB (Fig. 4b). Taken together, these findings further illustrate that CG is adjusted to accommodate the perturbation properties as AP MoS becomes asymmetric and ML MoS attenuates.

### *Baseline phase vs. Wash-out phase* – The effect of unannounced perturbation removal on anticipatory locomotor control

The persistence of CG was determined by comparing EW and LW with LB. Following the announced removal of the perturbation, participants walked with considerably lower step length_R_ and significantly higher step length_L_ compared to LB (Fig. 2b). Single support time_R,L_, were not significantly different from LB during EW (Table 2 for statistics). Nevertheless, the gait pattern was still characterized by spatiotemporal asymmetry during EW compared to LB. Furthermore, the step width was significantly decreased to a level lower than LB during EW. Throughout the wash-out phase participants increased step length_R_ (Fig. 2a), whereas step length_L_ remained relatively unchanged. Therefore, step length_L_ was still significantly higher whereas step length_R_ was not significantly different during LW than LB (Fig. 2b). Like step length, participants increased their single support time during the wash-out phase. Single support time_L_ was significantly higher during LW than LB, whereas single support time_R_ was not significantly different during LW compared to LB. Both step length and single support symmetry returned to LB level during LW, whereas step width remained significantly lower compared to LB. Overall, these results show that participants temporarily persist in displaying CG following the unannounced removal of the perturbation.

During the EW, AP MoS_R_ was significantly lower and AP MoS_L_ higher than LB (Fig. 3b). The ML MoS_R,L_ were lower during EW than LB (Fig. 4b), but only significantly for ML MoS_R_. Throughout the wash-out phase, AP MoS_R_ increased, and AP MoS_L_ remained relatively unchanged (Fig. 3a). Therefore, AP MoS_L_ was still significantly higher during LW compared to LB, whereas AP MoS_R_ was not significantly different from LB during LW (Fig. 3b). Furthermore, ML MoS_R,L_ decreased further during the wash-out phase (Fig. 4a) and was significantly lower during LW compared to LB (Fig. 4b).

## Discussion

In the present study, we addressed *if* and *how* anticipatory control of gait is adjusted to accommodate the requirements of a gait perturbation task. Warning participants about an upcoming perturbation initially resulted in a CG pattern that spontaneously extinguished over time. This temporary gait pattern was characterized by wider and shorter bilateral steps. Upon perturbation exposure, participants altered this generic strategy by taking shorter steps only with the perturbed leg and increasing the step length of the unperturbed leg. These were functional adjustments for the specific nature (slip) and side (right) of the perturbation. However, the (in)consistency of the perturbation sequence did not significantly affect the CG pattern. Following the unannounced removal of the perturbations, participants persisted in displaying CG temporarily, but this extinguished spontaneously over time. Overall, the present study shows that anticipatory control is functionally adjusted to accommodate the requirements of the task.

The expectation of an upcoming perturbation hazard was sufficient to trigger anticipatory responses. Previous studies have shown that young adults reduce their stride and step lengths when they are warned of a perturbation^11,13^. In line with this, we found that participants reduced their step lengths following a warning. In addition, participants increased their step width and reduced their single support times. Such anticipatory changes affect dynamic balance, in the sense that a shorter step enhances the stability against AP slips^13,17,18^, and a wider step or reduced single support times improves the stability against ML perturbations^17^. Accordingly, we found that anticipatory changes to enhance dynamic balance in the AP and ML direction were reflected in reduced AP MoS and increased ML MoS, respectively. Here, the dynamic balance was likely safeguarded in multiple planes as the features of potential perturbation were still uncertain to the participant. These anticipatory adjustments did not persist throughout the warning phase. This suggests that CG spontaneously extinguishes when the instruction (i.e. the warning) is not reinforced through exposure to the expected perturbation stimuli.

Anticipatory control was functionally adjusted to accommodate the perturbation properties through experience. Participants adjusted their gait to accommodate the nature of the task, by decreasing the step of the perturbed leg and increasing the step of the unperturbed leg. Since these slip-like perturbations accelerate the BoS relative to the CoM, a backward loss of balance needs to be arrested to ensure safe locomotion. Biomechanically, this can be effectuated by reducing the AP MoS^16^ through foot placement^17,31^ and reductions in gait speed^31^. As the gait speed was fixed, participants safeguarded their stability against AP slips by taking a shorter step with the perturbed leg. The reduced step length of the perturbed leg is consistent with findings reported previously^22,32^. In addition, we found that participants increased the step length of the unperturbed leg. This shows that the consistent side and nature of the perturbation became evident with experience and that this promoted task-specific adjustments. Such specificity is further substantiated by adjustments in the frontal plane. Initially, participants increased the ML MoS to safeguard against ML perturbations. However, as previous findings have shown that ML motion of the slipping foot is absent in simulated slip perturbations^33^, ML balance may not have been profoundly challenged. Therefore, the task-irrelevant increase in ML MoS strongly attenuated throughout the perturbation phase. Conjointly, these results show that CG is an adaptable and dynamic strategy. Anticipatory adjustments that are relevant for the present task requirements become dominant, whereas irrelevant adjustments are abandoned.

Inconsistency in the perturbation intensity did not affect anticipatory control as CG was not significantly different between groups. Previous work has shown that participants make anticipatory adjustments based on the condition or trial previously experienced^34^. In a study by Pavol & Pai^34^ participants were exposed to slip-perturbations during a sit-to-stand transition. By randomly alternating slip and non-slip trials, they showed that participants proactively improved stability against backward loss of balance following a slip trial while they improved stability against forward loss balance following a non-slip trial. In the present study, the intensity of consecutive perturbations was either slightly varied or kept consistent, but this did not yield different anticipatory control. The task requirements of consecutive perturbations may not have been distinctive enough due to moderate differences in perturbation intensity (i.e. 0.02 sec.) and high consistency in perturbation nature, side, and timing (i.e. shortly after foot strike). Hence, the employed strategy may have been sufficient to accommodate the imposed range of perturbation intensities. Alternatively, participants may simply have not been able to detect the moderate changes within the perturbation intensity, and as such did not display a different CG.

When the perturbations were removed, participants initially persisted in displaying CG. However, as the presence of the simulated slip was no longer reinforced through behavioural experience, swift and spontaneous extinction of CG occurred. The rate at which people learn this new context (i.e. extinction) may be related to the inter-perturbation interval during exposure. In conditioning studies, it has been shown that conditioned responses extinguish more slowly when the time between reinforcements with the conditioned stimulus is larger^35^. In the present study, the number of steps between consecutive perturbations was randomly varied between 5 and 14 strides. This relatively small inter-perturbation interval may have induced the swift rate of extinction during the wash-out phase. When the inter-perturbation interval is increased, people may learn to expect the perturbation longer and persist in displaying CG longer when the perturbations are removed. In addition, the persistence of CG may also depend on the self-perceived ability to maintain dynamic balance. As such, the extinction rate of CG may have the potential to provide an objective, behavioral measure to assess falls efficacy, a predictor of future fall risk^36^, in fall-prone populations.

Although the present study involves young adults these findings may have implications for fall-prevention training. This is an emerging paradigm that utilizes simulated perturbation exposure to improve reactive control of stability^7^. It has been shown that a combination of blocked and randomized block practice with simulated slips yields improved retention of proactive and reactive gait stability^37^. When motor learning principles like random practice and over-learning are not incorporated in slip training, only improvements in proactive stability are retained^38^. In the present study, we show that young adults almost instantaneously make perturbation specific, anticipatory adjustments to unburden the reactive control. However, this may reduce the effectiveness of the training and reduce the generalizability of trained reactive responses to daily life conditions. Arguably, a larger variety in perturbation properties may reduce the predominant reliance on anticipatory control and emphasize reactive control more. This may improve the retention of trained responses and increase its generalizability to daily life situations.

The present study addressed *if* and *how* anticipatory control is adjusted to accommodate the requirements of a gait perturbation task. Overall, the results indicate that both a slip warning and initial perturbation exposure induced a generic CG. However, this anticipatory gait strategy was recalibrated through experience with the requirements of the task. This led to the extinction of CG during the warning and wash-out phases but induced an increasingly more specific CG during the perturbation phase. The present results give insight into *how* anticipatory control of gait is recalibrated through behavioural experience with the task. These findings may aid in the development of targeted perturbation-based interventions and emphasize that CG in young adults does not allude to a one size fits all principle.

## Supplementary information

Supplementary information.

Supplementary information 2.
