# Supplementary material for: Anticipatory control of human gait following simulated slip exposure
Source: Sci Rep. 2020 Jun 15;10:9599. doi: 10.1038/s41598-020-66305-1 (PMC7295774; doi:10.1038/s41598-020-66305-1)
Supplement: Supplementary file 2 — Supplementary information 2. [file 41598_2020_66305_MOESM2_ESM.pdf]

Supplementary information

Anticipatory control of human gait following simulated slip exposure

Sander B. Swart[1*], Rob den Otter[1], Claudine J.C. Lamoth[1]

[1]*University of Groningen, University Medical Centre Groningen, Department of Human Movement Sciences, Groningen, The Netherlands*

[*]Corresponding author email: s.b.swart@umcg.nl

Supplementary table S1 - Perturbation characteristics, imposed and measured for the consistent group (N=10), pseudo-random group (N=10) and random group (N=10).

**Groups**	**Variability** *Mean (std)*	**Perturbation Duration (s)** *Mean (s.d.)*		**Perturbation Delay (s)** *Mean (s.d.)*	
	*Trial to trial**	*Imposed***	*Additional****	*Imposed*	*Measured*
Consistent	0 (0.0)	0.280 (0.0)	0.17 (0.023)	0.05 (0.0)	0.081 (0.056)
Pseudo-random	0.020 (0.020)	0.282 (0.114)	0.17 (0.027)	0.05 (0.0)	0.077 (0.048)
Random	0.123 (0.158)	0.282 (0.114)	0.17 (0.022)	0.05 (0.0)	0.061 (0.023)

* Trial to trial variability is calculated by taking the mean of differences between subsequent imposed perturbation durations

**Imposed duration reflects the averaged imposed perturbation duration of all perturbations

***Additional duration reflects the difference between imposed and total calculated duration

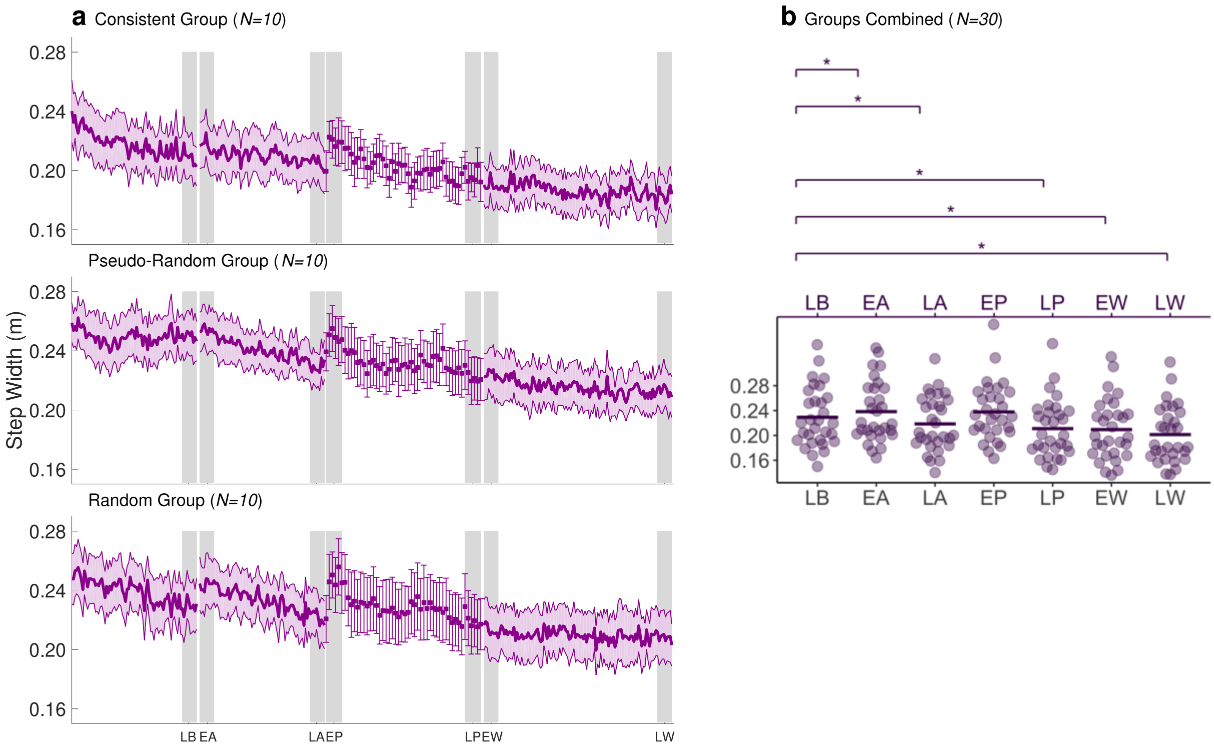

Supplementary figure S2 - Step Width during the experiment. *(a)* Step width during the experiment is shown for the consistent (top) (N=10), pseudo-random (middle) (N=10) and the random group (bottom) (N=10). Step width was averaged in bins of 5 strides during the baseline, warning and wash-out phase. During the perturbation phase, step width of the unperturbed strides between two consecutive perturbations were averaged. Shaded areas and whiskers around the mean represent the standard error. Grey shaded blocks represent the different phases throughout the experiment: late baseline (LB), early anticipation (EA), late anticipation (LA), early perturbation (EP), late perturbation (LP), early wash-out (EW) and late wash-out (LW). *(b)* Individual data points of all participants (N=30) during the experimental phases. Black and red horizontal lines, respectively, represent the group mean. Significant post hoc comparisons for the within factor PHASE are shown with the asterisk.

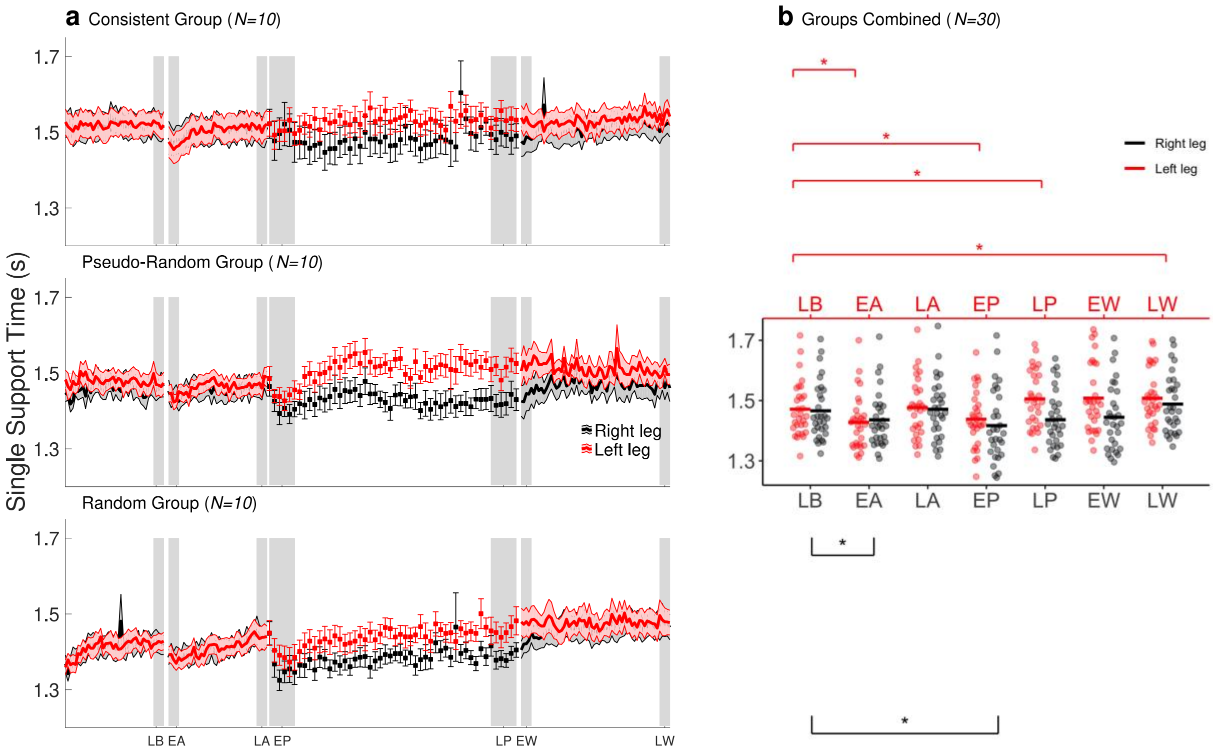

Supplementary figure S3 - Single Support time during the experiment. *(a)* Normalized single support times [26] during the experiment is shown for the consistent (top) (N=10), pseudo-random (middle) (N=10) and the random group (bottom) (N=10). Left and right single support times were averaged in bins of 5 strides during the baseline, warning and wash-out phase. During the perturbation phase, the left and right single support times of the unperturbed strides between two consecutive perturbations were averaged. Shaded areas and whiskers around the mean represent the standard error. Grey shaded blocks represent the different phases throughout the experiment: late baseline (LB), early anticipation (EA), late anticipation (LA), early perturbation (EP), late perturbation (LP), early wash-out (EW) and late wash-out (LW). *(b)* Individual data points of all participants (N=30) during the experimental phases. Black and red horizontal lines, respectively, represent the group mean. Significant post hoc comparisons for the within factor PHASE are shown with the asterisk.
